# Diabetes and prediabetes prevalence through a community-based screening initiative in Alqunfudah, Saudi Arabia

**DOI:** 10.2144/fsoa-2023-0208

**Published:** 2024-05-15

**Authors:** Saleh Ahmed Alshaikhi, Ahmed Madini Alamri, Ibrahim Yahya Alzilai, Ageel Ahmad Alghanimi, Ali Mohammad Alrufaidi, Ahmed Mohammed Alrufaidi, Ahmed Elsayed Bader, Ahmed Abushaisha Abdelmoniem, Ayoub Ali Alshaikh, Omar Ahmed Alshaikhi, Mohammed Ahmed Alshaikhi, Ramy Mohamed Ghazy

**Affiliations:** 1 Ministry of Health, Saudi Arabia; 2 Department of Family & Community Medicine, College of Medicine, King Khalid University, Abha, Saudi Arabia; 3 Medical student in King Saud bin Abdulaziz University for Health Sciences, Saudi Arabia; 4 Tropical Health Department, High Institute of Public Health, Alexandria University, Alexandria, 21561, Egypt

**Keywords:** community-based participatory research, community engagement, obesity, prediabetes and diabetes epidemic, Saudi Arabia, screening

## Abstract

**Aim:** This study aimed to identify prediabetic and diabetic patients using fasting blood sugar in Alqunfudah, Saudi Arabia. **Patients & methods:** Multistage stratified random sampling technique was used to recruit study participants aged 18 years and older. We measured anthropometric measures like waist circumference and body mass index. **Results:** A total of 332 participants were included in this study, 52.4% were female, 45.2% aged >50 years, 89.8% were Saudi, and 19.0% had been diagnosed with hypertension. Nearly a third (36.1%) of the participants were diagnosed with Type 2 diabetes mellitus and 28.3% had impaired fasting glucose. Age and hypertension were significant predictors of diabetes. **Conclusion:** Early detection and intervention are crucial to reducing the diabetes epidemic in Saudi Arabia.

## Plain language summary

Type 2 diabetes mellitus (T2DM) is a widespread health concern. Saudi Arabia is among the countries with the highest prevalence of diabetes. We conducted a cross sectional study in Alqunfudah, Saudi Arabia, to identify apparently healthy individuals who had undiagnosed T2DM and prediabetics. Through community engagement, we carefully selected apparently health participants from the general population in Alqunfudah, regardless of age or nationality. We measured various health indicators like waist circumference, body mass index, blood pressure, and fasting blood sugar levels. We included 332 participants in our study. Among them, slightly more than half were women, and a large sector was over 50 years old. Most of the participants were Saudi, and some had been diagnosed with hypertension. When we measured fasting blood sugar levels, we found that a considerable portion had T2DM, while others had impaired fasting blood sugar (prediabetic), indicating a risk of developing T2DM. Additionally, our analysis revealed that factors such as age and hypertension were associated with the diagnosis of T2DM. Our study highlights a significant prevalence of asymptomatic T2DM and prediabetes among the general population in Alqunfudah, Saudi Arabia. Early detection and intervention are critical for managing these conditions effectively. We recommend that local health authorities consider implementing screening programs for T2DM and prediabetes through community-based approaches to identify and assist people at risk.

## Tweetable abstract

Study in Alqunfudah, Saudi Arabia uncovers a concerning prevalence of diabetes & prediabetes in the general population. Early detection is vital. Urging local health authorities to initiate screening programs for early intervention using community engagement-based approach. #Diabetes #PublicHealth. More support is needed. #Obesity #community engagement

## Abstract (plain language summary)

High prevalence of diabetes and prediabetes found in Alqunfudah, Saudi Arabia, with nearly 36.1% of participants diagnosed with diabetes and 28.3% having impaired fasting blood glucose.

Age and hypertension, in particular, emerge as significant predictors of diabetes, emphasizing the importance of early detection, risk assessment and intervention strategies.

Urgent need for widespread diabetes prevention efforts, including lifestyle interventions and public health campaigns promoting healthy habits.

Implementation of screening programs to identify at-risk individuals and provide early intervention and education about diabetes risk factors.

Enhancements in healthcare infrastructure, research, cultural sensitivity and collaboration among stakeholders are essential to effectively tackle the diabetes mellitus epidemic in the region.

The findings highlight the critical need for immediate action in the form of diabetes prevention initiatives. This includes encouraging people to live healthier lives through dietary changes and increased physical activity.

It is critical that public health programs that raise diabetes awareness among both Saudi and non-Saudi residents of the region are developed. These initiatives should prioritize education and outreach to marginalized communities.

Continued research into diabetes epidemiology and causal variables in the region is critical. Regular surveillance efforts are also required to assess intervention efficacy and change methods over time.

All approaches must be culturally sensitive. Programs that are tailored to local cultural norms and beliefs will result in greater participation and outcomes.

To improve diabetes care, telemedicine and remote monitoring services, particularly in rural areas, should be developed. This will enable more rapid access to care and support.

Collaboration between healthcare organizations, government agencies, and community groups is critical. Partnerships can increase the overall impact of diabetes management efforts by facilitating resource sharing and knowledge exchange.

Long-term follow-up studies are essential for assessing intervention efficacy, monitoring changes in diabetes prevalence and informing future policies and actions.

Diabetes mellitus (DM) is a global health problem, affecting roughly 537 million adults aged 20 to 79, or one out of every ten people. This alarming figure is projected to escalate to 643 million by the year 2030 and a staggering 783 million by 2045 [1]. Worryingly, more than 75% of adults with DM reside in low- and middle-income countries, highlighting the unequal burden of this disease. The impact of DM extends beyond its prevalence, as it was responsible for 6.7 million deaths in 2021, a life lost every 5 seconds [2]. Type 2 diabetes mellitus (T2DM) accounts for 90% of all cases of DM and is characterized by insufficient insulin secretion by pancreatic islet cells, tissue insulin resistance (IR) and an inadequate compensatory insulin secretory response [3,4]. Numerous risk factors, both modifiable and nonmodifiable, influence the onset and progression of T2DM. Obesity, inactivity, unhealthy eating habits, hypertension, and smoking are all modifiable risk factors. Nonmodifiable risk factors include age, family history, ethnicity, and genetic factors [^5-7^]. In fact, dysglycemia, or inadequate regulation of blood glucose levels, is a term used to describe both T2DM and prediabetes, the latter is a condition that precedes diabetes. Compared with people with normal glucose tolerance and blood glucose levels, those with prediabetes have a five to ten-times greater annual risk of developing T2DM [8]. But there is substantial evidence that lifestyle modification can delay the onset of T2DM from prediabetes. The incidence of T2DM can be reduced by half by significant lifestyle changes with long-term effects of 34–45% lower incidence over a period of 9 to 23 years of follow-up [^9-11^]. Moreover, early glycemic management lowers a diabetic's risk for short- and long-term macrovascular problems, as well as mortality [12].

The prevalence of DM is second highest in the Middle East and seventh worldwide in Saudi Arabia [13]. It is estimated that over 7 million people are diabetic, with nearly 3 million having pre-diabetes [14]. A comprehensive epidemiological health survey was carried out in Saudi Arabia, targeting adults aged 30–70 years who lived in selected households. Of the 16,917 survey participants, 4004 were diagnosed with T2DM, representing approximately 23.7% of the studied population [15]. However, based on other studies, Saudis have been found to have higher prevalence rates of T2DM, ranging from 26.0 to 61.8% [16,17]. T2DM in Saudi Arabia has risen tenfold in the past three decades, leading to high mortality, morbidity, cardiovascular complications, poor health and lower quality of life, becoming a significant medical issue [14]. In 2016, the number of Saudi citizens affected by cardiovascular diseases (CVDs) amounted to 201,300, encompassing 149,600 adults diagnosed with ischemic heart disease and 51,700 with cerebrovascular disease. It is worth noting that CVDs is responsible for more than 45% of all recorded fatalities [18,19].

T2DM operates insidiously, with over 50% of the global affected population are unaware of their condition until symptoms or complications manifest. This silent progression underscores the critical importance of T2DM screening [12]. Recognized as a disease with potential for early detection, clinical guidelines, advocate for the screening of T2DM [20]. The primary objective of screening is to identify asymptomatic individuals with DM promptly, enabling early therapeutic intervention and positively influencing the course of the disease [21].

Chronic diseases have social and political causes, as well as physiological mechanisms. However, most healthcare professionals and organizations address chronic disorders on an individual or intrapersonal level. It is necessary to adopt a more comprehensive strategy to address the root causes of these chronic diseases, including community participation in the definition of the problem and the formation of partnerships to develop and implement effective and long-term therapies and management approaches [22]. By involving the community in research, communities may be better able to address their own health needs and concerns related to health disparities while also ensuring that researchers are aware of local priorities. However, some researchers have little familiarity or expertise in successful community engagement strategies. Furthermore, peer review panels can only use a limited amount of help when reviewing research proposals that involve communities [23]. This study hypothesized that involving the community actively in research could enhance the diagnosis and management of prediabetic and diabetic patients in Saudi Arabia, ultimately leading to improved health outcomes and a reduction in health disparities. The primary objective of the study was to identify individuals with prediabetes and diabetes among healthy adults in the Alqunfudah community in Saudi Arabia using fasting blood sugar as a diagnostic marker.

## Materials & methods

### Study design & study setting

This study was conducted in Alqunfudah, Saudi Arabia, and used a quantitative observational analytical cross-sectional design. Alqunfudah is located in the Makkah Province, about 300 km south of Jeddah. It is the fourth-most populous city in Makkah Province. The governate has an estimated area of 5195 km^2^, accounting for approximately 3.65% of the region's total area and ranking ninth in terms of area among the governorates in the region. Alqunfudah is also a significant Red Sea seaport, with an estimated population of over 297,000 people living in urban and coastal villages, accounting for approximately 2.8% of the province's population.

### Study population

We used G*Power software to calculate the required sample size for our study. Based on the following assumptions: a significance level (alpha) of 0.05, size effect of 0.1, a statistical power of 95%, and a prevalence of undiagnosed diabetes mellitus at 58.8% [24], the minimum required sample size was computed to be 304 participants. The target population for this study comprised apparently healthy individuals residing in Saudi Arabia, including both Saudi and non-Saudi nationals. We specifically included adults aged 18 years and above in our study. However, we excluded individuals with confirmed DM and those with mental disorders or communication problems.

### Sampling technique

**Figure 1.**
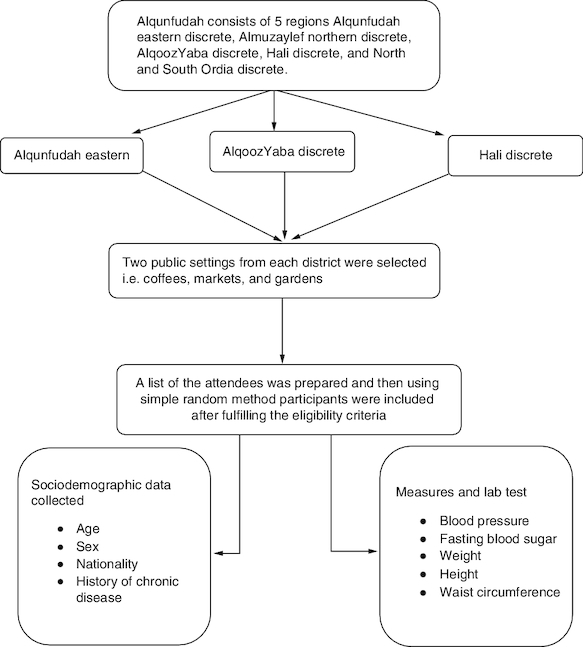
The study sample selection procures and study procedures.

A multistage stratified random sampling technique was used to recruit study participants. Alqunfudah consists of five regions Alqunfudah eastern discrete, Almuzaylef northern discrete, Alqooz Yaba discrete, Hali discrete, and North and South Ordia discrete. Three districts were randomly selected. Two public settings from each district were selected randomly to conduct the screening program, including coffees, markets, and gardens. Participants were recruited from each selected setting using random selection method. A list of the attendees was prepared and then using simple random method participants were included after fulfilling the eligibility criteria. For each selected setting, we posted an announcement on the wall explaining the study objectives and the duration of fasting (6–8 h) required to measure fasting blood glucose as an initiative to diagnose T2DM (Figure 1).

### Study questionnaire & data collection

To investigate factors associated with the prevalence of T2DM in Alqunfudah city, a pre-designed structured questionnaire was employed. The questionnaire comprised three distinct sections, covering relevant aspects related to T2DM. Before its use, the questionnaire underwent a pilot phase involving 15 subjects. This pilot aimed to assess the clarity of the wording and the time required to complete the questionnaire. Responses obtained during this pilot phase were excluded from the subsequent analysis to ensure the reliability and validity of the data collected for the main study. Participants were prompted to indicate their gender, with choices for ‘female’ or ‘male’. Age was categorized into different groups, ranging from ‘18–20’ to ‘>50'. The health-related variables include questions about hypertension, with responses of ‘no' or ‘yes', and bronchial asthma, with the same response options. Lastly, participants were asked to specify their nationality as ‘non-Saudi' or ‘Saudi'.

The second section of the questionnaire aimed to determine if the participant has any pre-existing chronic diseases. Finally, body measures; BMI was calculated by dividing an individual's weight in kilograms by the square of his height in meters (BMI = weight in kg/[height in m]^2^). The WHO classifies BMI into several categories to assess an individual's weight and health status. These categories include underweight, defined as a BMI less than 18.5, normal weight, with a BMI ranging from 18.5 to 24.9, overweight, where the BMI falls between 25.0 and 29.9, and various classes of obesity: class I (BMI 30.0–34.9), class II (BMI 35.0–39.9) and class III (BMI ≥40.0). Waist Circumference (WC): The measurement was taken at the midpoint between the lower margin of the last palpable rib and the top of the iliac crest, using a non-stretchable measuring tape. Blood pressure measurement using a sphygmomanometer. Fasting blood glucose level was typically measured in mg/dl using Beurer Glucometer. This tool showed the highest accuracy compared with other tool [25]. The diagnostic criteria for diabetes and prediabetes, as per the American Diabetes Association (ADA), are:

- Normal: fasting blood glucose <100 mg/dl (5.6 mmol/l);
- Prediabetes (impaired fasting glucose): fasting blood glucose 100–125 mg/dl (5.6–6.9 mmol/l);
- Diabetes: fasting blood glucose ≥126 mg/dl (7.0 mmol/l).

All those diagnosed with diabetes and prediabetes were referred to the nearest primary health unit to confirm diagnosis and for further treatment. Non-diabetics were instructed on how to prevent diabetes and adopt a healthy lifestyle.

### Ethical apporval & consent

The study objectives were effectively communicated to all participants. Prior to the start of the study, informed written consent was obtained from all study participants. The ethical approval for this study was granted by the Research Ethics Board of the University of King Khalid (IRB: ECM#2023-2013) in adherence with the ethical principles outlined in the Declaration of Helsinki. Study participants were duly notified that they had the option to withdraw their participation from the study at any time. In addition, they were informed that the information collected is confidential and restricted in access, limited solely to the principal investigator and the statistician. It was explicitly conveyed that these data would not be used for any other publication.

### Statistical analysis

A systematic data quality check was performed to ensure the accuracy, completeness and reliability of the research data. Statistical analysis was carried out using the statistical package of social science (SPSS) software version 27. Categorical variables were presented as frequencies and percentages. To compare two independent numerical variables, Pearson's chi-square test was used. Binary logistic regression analysis was performed to identify predictors of diabetes, including variables such as age, sex, nationality, and BMI. The odds ratio (OR) and the corresponding 95% confidence intervals (CIs) were calculated to assess the association between these factors and the likelihood of having diabetes. The p-value, which signifies the level of statistical significance, was set at a threshold of less than 0.05.

## Results

This study involved 332 participants, maintaining a gender balance with 52.4% female and 47.6% male participants. The age distribution revealed that nearly half of the participants (45.2%) were above 50 years old, with the 40–50 years age group accounting for 20.8% of the sample. The majority of participants were Saudi nationals (89.8%), while 10.2% were non-Saudi. In terms of medical conditions, approximately four-fifths of the participants (81%) did not have hypertension, and an overwhelming majority (99.1%) did not have bronchial asthma (Table 1).

**Table 1.** Sociodemographic characteristics of the studied population.

Variables	Level	Frequency	%
Sex	Female	174	52.4
	Male	158	47.6
Age (years)	^18-20^	13	3.9
	^20-30^	47	14.2
	^30-40^	53	16.0
	^40-50^	69	20.8
	>50	150	45.2
Nationality	Non-Saudi	34	10.2
	Saudi	298	89.8
Hypertension	No	269	81.0
	Yes	63	19.0
Bronchial asthma	No	329	99.1
	Yes	3	0.9

The studied population had a mean BMI of 28.3 ± 6.5, with a range of values from 9.5 to 28.3. The mean WC was 86.9 ± 12.0 ranging from 27.0 to 146.0 cm. Systolic blood pressure averaged at 132.3 ± 21.8 mmHg, with a range from 69.0 to 236.0 mmHg. Diastolic blood pressure had a mean of 75.5 ± 11.5 mmHg, ranging from 50.0 to 125.0 mmHg. Fasting blood sugar levels had a mean of 126.3 ± 53.0 mg/dl, with values ranging from 61.0 to 455.0 mg/dl (Table 2).

**Table 2.** Shows the body measures and blood pressure of the population studied.

Measurement	Minimum	Maximum	Mean	Standard deviation
Body mass index	9.5	28.3	28.3	6.5
Waist circumference (cm)	27.0	146.0	86.9	12.0
Systolic blood pressure (mmHg)	69.0	236.0	132.3	21.8
Diastolic blood pressure (mmHg)	50.0	125.0	75.5	11.5
Fasting blood glucose (mg/dl)	61.0	455.0	126.3	53.0

**Figure 2.**
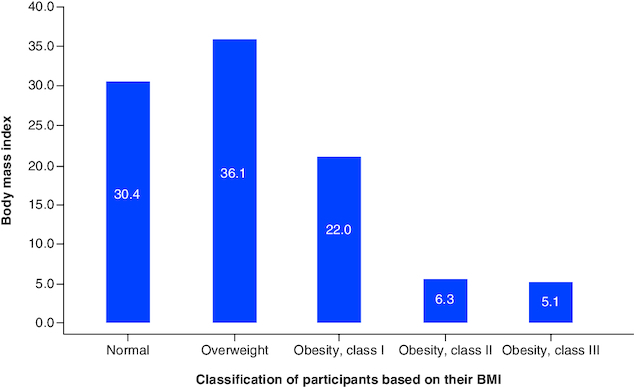
The distribution of the studied sample based on their BMI, the mean score of the QOL domains.

Figure 2 shows the distribution of the studied population based on the normal BMI category. Interestingly, only 30.4% had a normal BMI, while 29.6% of the participants experienced either overweight (36.1%), or obesity (33.4%).

Table 3 presents the correlation between fasting blood sugar and various studied variables, including systolic blood pressure, diastolic blood pressure, height, weight, WC and BMI. FBS had a significant positive correlation with systolic blood pressure (r = 0.215) and WC (r = 0.205), both at the 0.01 significance level (two-tailed). Fasting blood sugar also had a significant positive correlation with height (r = 0.197) at the 0.01 significance level (two-tailed).

**Table 3.** Correlation between fasting blood sugar and different studied variables.

Variables		FBS	SBP	DBP	Height	Weight	WC	BMI
FBS	r	1	.215^†^	0.05	-0.006	.205^†^	0.072	.197^†^
	*p*		0.0001	0.3690	0.9100	0.0001	0.1910	0.0001
Systolic blood pressure (SBP)	r		1	.499^†^	-0.006	.266^†^	0.09	.298^†^
	*p*			0.0001	0.9090	0.0001	0.1050	0.0001
Diastolic blood pressure (DPB)	r			1	0.081	.185^†^	-0.006	.161^†^
	*p*				0.142	0.001	0.912	0.003
Height	r				1	.345^†^	0.079	-.239^†^
	*p*					0.0001	0.148	0.0001
Weight	r					1	.284^†^	.789^†^
	*p*						0.0001	0.0001
Waist circumference	r						1	.241^†^
	*p*							0.0001

DBP: Diastolic blood pressure; FBS: Fasting blood sugar; p: Significance; r: Correlation coefficient; SBP: Systolic blood pressure; WC: Waist circumference.

† Correlation is significant at the 0.01 level (2-tailed).

**Figure 3.**
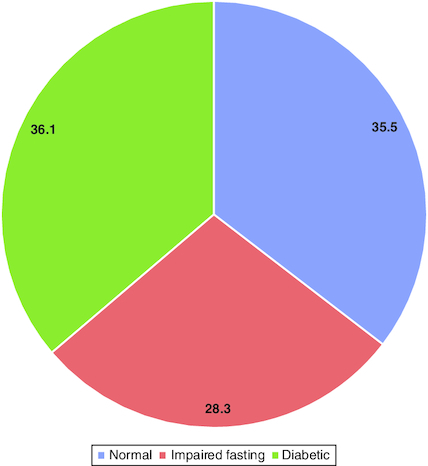
Glycemic status of the screened population based on fasting blood glucose.

Prevalence of diabetes based on fasting blood glucose, almost a third (36.1%) of the participants were diagnosed with T2DM. While 28.3% had impaired fasting glucose (Figure 3).

We examined the distribution of participants across various demographic factors to assess their association with diabetic status. Among females and males, the prevalence of T2DM was quite similar, with 36.78% for females and 35.44% for males. The difference between the two groups was not statistically significant (p = 0.800). In terms of nationality, 32.35% of non-Saudi individuals were diagnosed with T2DM, while a slightly higher prevalence of 36.58% was found among Saudi participants. However, this variation was not statistically significant. An important observation from the study was the notable correlation between the diagnosis of T2DM and advancing age. The prevalence of T2DM varied significantly across age groups. The lowest rates were found in the 15–20 and 20–30 years age groups, registering at 7.69 and 6.38%, respectively. In contrast, the highest prevalence was evident among adults aged over 50 years, reaching 54.67% (p < 0.001). Additionally, we explored the prevalence of T2DM in participants with and without bronchial asthma. Interestingly, the prevalence was approximately twice as high among participants with bronchial asthma (66.67%) compared with those without asthma (35.87%). Nonetheless, this difference was not statistically significant (p = 0.269) (Table 4).

**Table 4.** Factors associated with incidence of undiagnosed diabetes.

Studied variables	Variable	Total		Diabetes				p-value
		N	%	Non diabetic (n,%)		Diabetic (n, %)		
Gender	Female	174	52.40%	110	63.22%	64	36.78%	0.800
	Male	158	47.60%	102	64.56%	56	35.44%	
Nationality	Non-Saudi	34	10.20%	23	67.65%	11	32.35%	0.627
	Saudi	298	89.80%	189	63.42%	109	36.58%	
Age (in years)	^15-20^	13	3.90%	12	92.31%	1	7.69%	<0.001
	^20-30^	47	14.20%	44	93.62%	3	6.38%	
	^30-40^	53	16.00%	42	79.25%	11	20.75%	
	^40-50^	69	20.80%	46	66.67%	23	33.33%	
	>50	150	45.20%	68	45.33%	82	54.67%	
Bronchial asthma	No	329	99.10%	211	64.13%	118	35.87%	0.269
	Yes	3	0.90%	1	33.33%	2	66.67%	

**Figure 4.**
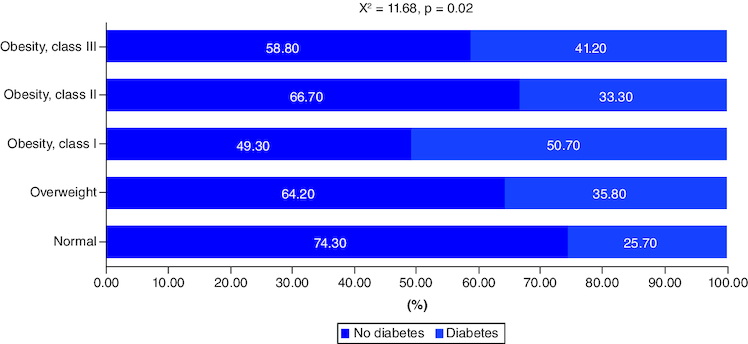
Association between obesity and diabetes mellitus.

The association between obesity and T2DM is visually depicted in Figure 4. The findings revealed a substantial correlation, with significantly higher rates of diabetes observed among individuals with higher BMI. Specifically, 74.30% of those with normal body weight did not have T2DM, whereas this percentage was lower for those classified as overweight (64.2%), obese class I (49.30%), obese class II (66.7%), and obese class III (58.8%). The chi-square test (X^2^ = 11.68; p = 0.020) indicates a statistical significance of this association.

Table 5 presents a comparison of various measures between diabetic and non-diabetic. The table revealed a statistically significant difference in systolic blood pressure between participants diagnosed with T2DM (140.13 ± 22.02 mmHg) and those without T2DM (127.84 ± 20.41 mmHg), t = 5.10 and p-value <0.0001. Similarly, diastolic blood pressure differed significantly between diabetics (78.13 ± 1.6 mmHg) and non-diabetics (74.00 ± 11.15 mmHg), t-statistic = 3.13 and p-value = 0.002. The table did not show statistically significant differences in WC between diabetics (87.54 ±13.26 cm) and non-diabetics (86.52 ± 11.27 cm), t = 0.74 and p-value = 0.458. BMI values for diabetics was (29.62 ± 6.52) and non-diabetics was ( 27.49 ± SD: 6.40) also exhibit a statistically significant difference (t statistic = 2.90 and p-value = 0.004).

**Table 5.** Different measures across diabetic and non-diabetic participants

Group Statistics	Diabetic		Non-diabetic		t	df	*p*-value
	Mean	SD	Mean	SD			
Systolic blood pressure	140.13	22.02	127.84	20.41	5.10	326	<0.0001
Diastolic blood pressure	78.13	11.66	74.00	11.15	3.13	236.58	0.002
Waist circumference	87.54	13.26	86.52	11.27	0.74	330	0.458
Body mass index	29.62	6.52	27.49	6.40	2.90	330	0.004

df: Degree of freedom; SD: Standard deviation; t: T statistics.

Table 6 presents the results of a binary logistic regression analysis that evaluated the association between various variables and T2DM. Age was a highly significant predictor of T2DM as indicated by the Wald statistic of 16.11 and a highly significant p-value (0.003). Hypertension was a significant predictor of T2DM, with a Wald statistic of 25.44 and a highly significant p-value (0.0001). The OR value of 0.16 (95% CI: 0.08–0.33) suggests that individuals with hypertension have significantly higher odds of having T2DM (Table 6).

**Table 6.** Binary logistic regression of variables associated with diabetes.

Studied variables	S.E.	Wald	p-value	OR	95% CI	
					Lower	Upper
**Constant**	1.94	1.96	0.161	15.226		
Hypertension	0.36	25.44	**0.0001**	0.16	0.08	0.33
Waist circumference	0.01	0.01	0.913	1.00	0.98	1.02
Age category		16.11	**0.003**			
15–20 years	0.33	1.81	0.179	1.56	0.82	3.00
20–30 years	1.09	1.75	0.187	0.24	0.03	2.00
30–40 years	0.69	6.51	0.011	0.17	0.05	0.67
40–50 years	0.44	1.11	0.292	0.63	0.26	1.49
Bronchial asthma	1.48	2.04	0.153	0.12	0.01	2.19
Body mass index		2.93	0.570			
Normal	0.65	0.00	0.956	0.97	0.27	3.43
Overweight	0.62	0.14	0.705	1.27	0.37	4.31
Obesity, Class I	0.64	0.72	0.397	1.71	0.49	5.94
Obesity, Class II	0.78	0.04	0.833	0.85	0.19	3.90

Bold p-values less than 0.05 are significant.

OR: Odds ratio.

## Discussion

In this study we aimed to implement the community-based research to screen normal healthy adults in Alqunfudah, Saudi Arabia for T2DM and prediabetes. We recruited the population from common community settings such as coffee gardens, and markets. We found that nearly one third (36.1%) of the participants were diagnosed with T2DM while 28.3% had impaired fasting blood glucose. Age was a highly significant predictor of T2DM with different age categories (15–20, 20–30, 30–40, 40–50) had varying odds ratios. Hypertension was a significant predictor of T2DM, as well.

### Community engagement

Our study suggests that community engagement influences the provision of appropriate diabetes screening services and utilization. The pharmacist and primary care unit should emphasize community engagement in other DM prevention services. The approach of community engagement was proved to be effective in diabetes screening and management of T2DM [26,27]. This approach was also effective in management of many chronic diseases like bronchial asthma [28], hypertension [29] and obesity [30].

### Screening tool for diabetes mellitus

In this study we preferred to depend on a single most visible and sensitive test to diagnose T2DM. The best cut-off for fasting blood glucose was calculated to be 104 mg/dl based on prior meta-analysis findings, with a sensitivity of 82.3% (95% CI: 74.6–88.1%) and specificity of 89.4% (95% CI: 85.2–92.5%). The pooled sensitivity, specificity, positive (LR+) and negative likelihood ratio (LR-) for the diagnosis of diabetes with HbA1c (6.5%; venous sample; n = 17 studies) were 50% (95% CI: 42–59%), 97.3% (95% CI: 95.3–98.4), 18.32% (95% CI: 11.06–30.53), and 0.51% (95% CI: 0.43–0.60). As a result, for the community screening, we chose fasting blood sugar [31]. However, other studies use HbA1C as a screening tool or both [32,33]. Davidson *et al.*, found that HbA1c is an effective screening tool for DM in a community-based study involving 1542 African–American and Latino participants aged 40 years and older [32]. The 2011 Korea National Health and Nutrition Examination Survey revealed that relying solely on fasting plasma glucose as a diagnostic criterion may lead to an underestimation of both diabetes and prediabetes prevalence. When HbA1c was included as an additional diagnostic test, the prevalence of DM increased from 10.5 to 12.4%, and the prevalence of prediabetes increased from 19.3 to 38.3% [33].

### Prevalence of diabetes & prediabetes

In this survey, we found that 28.3% of the participants were prediabetic. A lower prevalence was reported in a study conducted in Al Bahah, Saudi Arabia. The authors found that 20% of adults have prediabetes, with a significant association with obesity, hypertension, and a family history of T2DM [34]. According to Saudi Arabia's National Survey of Health Information, male prevalence of prediabetes was 17% and female prevalence was 15.5% [35]. A study of 1420 residents of Jeddah aged 18 years and older found a prevalence of prediabetes at 9.0%, and a prevalence of diabetes at 12% with a higher rate in men than in women [36]. The progression of prediabetes can vary; a Saudi Arabian study of 92 individuals who were observed for 3 years found that 76.08% of them remained in the prediabetic range, while 16.4% regressed to a normal glycemic condition and 7.6% advanced to the diabetic range [27]. On the other hand, a lower prevalence of diabetes and prediabetes was observed among French population in a large screening program of 32,721 adults aged 40–70 years. The prevalence was 8.1% for prediabetes and 2.3 for T2DM. The main identified risk factors were older age, male sex, obesity and overweight [37]. Similarly, on World Health Day 2016, a nationwide screening in urban areas was conducted to screen Bengalis for T2DM. A total of 13,564 individuals were screened. The screening revealed a 7.5% age-standardized prevalence of diabetes using a blood glucose cut-off point of 11.1 mmol/l [38]. Our findings strongly support the Saudi population's increased risk of undiagnosed diabetes, emphasizing the critical need for the implementation of a comprehensive public health strategy. In Saudi Arabia, this plan should priorities efficient screening, diagnosis and treatment of diabetes and prediabetes. The recognition of this increased risk emphazises the importance of proactive measures to identify and manage diabetes cases early, ultimately contributing to the improvement of the country's overall public health.

### Prevalence of obesity

In this study 69.6% of the participants were overweight or obese. According to a study conducted in the Aseer region of Saudi Arabia, 33.6% of the 512 participants were overweight, and 20.5% were obese. The main determinants of obesity were female gender, age 12–17 years, place of birth, family size of more than eight members, family monthly income of more than 20,000 Saudi Riyal, being a smoker, experiencing social stress, engaging in physical activity less than three-times per week, and engaging in physical activity more than three times per week [39]. Another study in Makkah found that 32.8% of the population was overweight (41% of men and 28.9% of women), while 23% of the population was obese (males 23.1% and females 24.2%) [40]. This data indicates the high prevalence of obesity in Saudi Arabia. It worthy to note that the prevalence of diabetes was significantly higher among obese participant in bivariate analysis. This significant difference was not retained in the multivariate analysis. According to research, the chance of being diabetes is double or more among overweight and obese people compared with non-overweight people in all categories. A unit increase in BMI increases the probability of developing diabetes by roughly 1.5% among overweight and obese people [30].

### Aging & diabetes mellitus

We found a significant association between increasing age and DM. A similar finding was reported in previous studies [27,31]. As people age, T2DM and prediabetes are more common. The following are the two most significant causes of hyperglycemia: age-related declines in insulin production and increased insulin resistance caused by changes in body composition and sarcopenia [41].

### Hypertension & diabetes

Hypertension and T2DM are common comorbidities, with hypertension twice as common in diabetes patients and often exhibiting insulin resistance. CVDs are the leading cause of morbidity and mortality in diabetes, exacerbated by hypertension. Common mechanisms include upregulation of the renin–angiotensin–aldosterone system, oxidative stress, inflammation and immune system activation [42]. In this study, hypertension was a significant determinant of the diagnosis of DM. Similarly, a study analyzing blood pressure changes during hypertension development in patients with and without DM found that DM at baseline was a significant predictor of hypertension, while hypertension at baseline was an independent predictor of T2DM. The study concluded that hypertension and T2DM track each other over time, the transition from normotension to hypertension is characterized by a sharp increase in BP values, and insulin resistance is a common feature of both conditions [33].

### Strengths & limitations

One of the main points of strength of this survey is using the community-based approach to diagnose T2DM. In addition, we used a random sampling method that's ensure the internal validity of the study findings. However, the cross-sectional design provides a snapshot of data at a single point in time. It may not capture the dynamic nature of health conditions and does not establish causality between variables. Some of the data collected, such as chronic disease status and health-related behaviors, is based on self-reporting by participants. This introduces the possibility of recall bias or social desirability bias, where participants may not provide completely accurate information. Finally, although focusing on Alqunfudah provided local insights, it may limit the generalizability of the findings to broader populations, as health characteristics can vary across other Saudi regions.

## Conclusion

Community engagement was successful in recruiting apparently healthy population for this screening program. The findings of our study revealed a high prevalence of T2DM among the studied participants, with nearly one third of the individuals diagnosed with T2DM. Additionally, 28.3% of participants exhibited impaired fasting glucose. Notably, age emerged as a highly significant predictor of T2DM. Increasing age was associated with increased incidence of T2DM. Furthermore, the presence of hypertension also emerged as a significant predictor of T2DM. These findings underscore the importance of early detection and intervention in managing DM and related risk factors within this population. Health authorities must institute comprehensive screening programs for the early detection of both diabetic and prediabetic individuals.
